# 7-Chloro-4-[(*E*)-2-(3,4,5-trimeth­oxy­benzyl­idene)hydrazin-1-yl]quinoline

**DOI:** 10.1107/S1600536812012755

**Published:** 2012-03-28

**Authors:** Marcelle de Lima Ferreira, Marcus V. N. de Souza, Solange M. S. V. Wardell, Edward R. T. Tiekink, James L. Wardell

**Affiliations:** a Instituto de Tecnologia em Fármacos–Farmanguinhos, FioCruz–Fundação Oswaldo Cruz, R. Sizenando Nabuco, 100, Manguinhos, 21041-250 Rio de Janeiro, RJ, Brazil; b CHEMSOL, 1 Harcourt Road, Aberdeen AB15 5NY, Scotland; c Department of Chemistry, University of Malaya, 50603 Kuala Lumpur, Malaysia; d Centro de Desenvolvimento Tecnológico em Saúde (CDTS), Fundação Oswaldo Cruz (FIOCRUZ), Casa Amarela, Campus de Manguinhos, Av. Brasil 4365, 21040-900 Rio de Janeiro, RJ, Brazil

## Abstract

In the title compound, C_19_H_18_ClN_3_O_3_, the r.m.s. deviation through the 23 non-H and non-meth­oxy atoms is 0.088 Å, indicating a planar mol­ecule with the exception of the meth­oxy groups. One meth­oxy group, surrounded on either side by the other meth­oxy groups, is almost normal to the benzene ring to which it is connected [C—O—C_ar_—C_ar_ torsion angle = 81.64 (15)°]. In the crystal, N—H⋯O, C—H⋯O and π–π inter­actions [between quinoline residues; centroid–centroid distance = 3.4375 (8) Å] link mol­ecules into a three-dimensional architecture.

## Related literature

For the biological activity, including anti-tubercular and anti-tumour activity, of compounds containing the quinolinyl nucleus, see: de Souza *et al.* (2009 ▶); Candea *et al.* (2009 ▶); Montenegro *et al.* (2012 ▶). For related structures, see: Howie *et al.* (2010 ▶); de Souza *et al.* (2010 ▶, 2012 ▶).
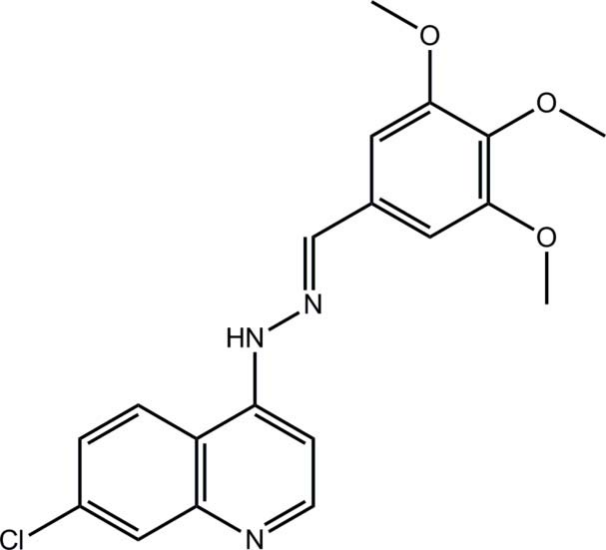


## Experimental

### 

#### Crystal data

C_19_H_18_ClN_3_O_3_
*M* *_r_* = 371.81
Orthorhombic, 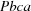

*a* = 7.6338 (2) Å
*b* = 15.5335 (4) Å
*c* = 28.7960 (7) Å
*V* = 3414.62 (15) Å^3^
*Z* = 8
Mo *K*α radiation
μ = 0.25 mm^−1^
*T* = 120 K
0.45 × 0.40 × 0.30 mm

#### Data collection

Bruker–Nonius Roper CCD camera on a κ-goniostat diffractometer
Absorption correction: multi-scan (*SADABS*; Sheldrick, 2007 ▶) *T*_min_ = 0.652, *T*_max_ = 0.746
22673 measured reflections
3899 independent reflections
3353 reflections with *I* > 2σ(*I*)
*R*_int_ = 0.040

#### Refinement

*R*[*F* ^2^ > 2σ(*F* ^2^)] = 0.036
*wR*(*F* ^2^) = 0.101
*S* = 1.02
3899 reflections
241 parameters
1 restraint
H atoms treated by a mixture of independent and constrained refinement
Δρ_max_ = 0.30 e Å^−3^
Δρ_min_ = −0.30 e Å^−3^

### 

Data collection: *COLLECT* (Hooft, 1998 ▶); cell refinement: *DENZO* (Otwinowski & Minor, 1997 ▶) and *COLLECT*; data reduction: *DENZO* and *COLLECT*; program(s) used to solve structure: *SHELXS97* (Sheldrick, 2008 ▶); program(s) used to refine structure: *SHELXL97* (Sheldrick, 2008 ▶); molecular graphics: *ORTEP-3* (Farrugia, 1997 ▶) and *DIAMOND* (Brandenburg, 2006 ▶); software used to prepare material for publication: *publCIF* (Westrip, 2010 ▶).

## Supplementary Material

Crystal structure: contains datablock(s) global, I. DOI: 10.1107/S1600536812012755/bt5858sup1.cif

Structure factors: contains datablock(s) I. DOI: 10.1107/S1600536812012755/bt5858Isup2.hkl

Supplementary material file. DOI: 10.1107/S1600536812012755/bt5858Isup3.cml

Additional supplementary materials:  crystallographic information; 3D view; checkCIF report

## Figures and Tables

**Table 1:** Hydrogen-bond geometry (Å, °)

*D*—H⋯*A*	*D*—H	H⋯*A*	*D*⋯*A*	*D*—H⋯*A*
N2—H2*n*⋯O3^i^	0.87 (1)	2.53 (2)	3.0349 (15)	118 (1)
C19—H19*B*⋯N1^ii^	0.98	2.48	3.3602 (18)	149

Symmetry codes: (i) 
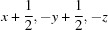
; (ii) 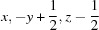.

**Fig. 1.**
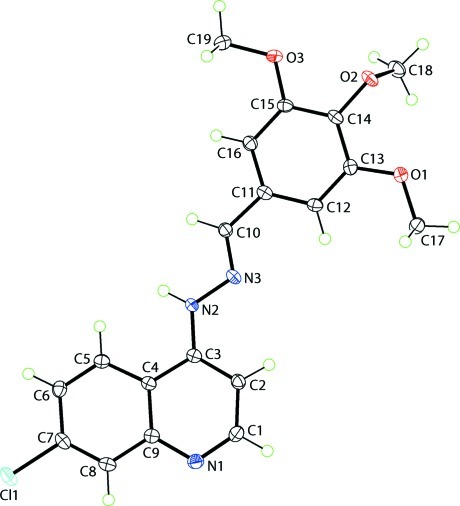
The molecular structure showing the atom-labelling scheme and displacement ellipsoids at the 50% probability level.

**Fig. 2.**
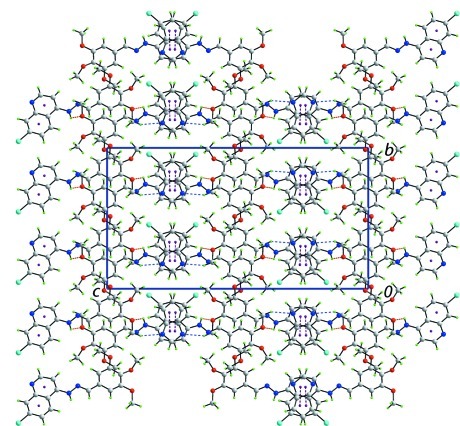
A view in projection down the a axis of the unit-cell contents of (I). The N—H···O, C—H···N and π···π interactions are shown as orange, blue and purple dashed lines, respectively.

## supplementary crystallographic information

### Comment

The title compound, (I), was investigated as part of on-going crystallographic investigations of arylaldehyde 7-chloroquinoline-4-hydrazone species (Howie *et al.*, 2010; de Souza *et al.*, 2010; de Souza *et al.*, 2012). The structural studies complement biological studies which show these hydrazones to possess a wide range of pharmacological activities such as anti-tubercular (Candea *et al.*, 2009) and anti-tumour (Montenegro *et al.*, 2012) activities, which are ascribed to the presence of the quinoline nucleus (de Souza *et al.*, 2009).

In (I), Fig. 1, with the exception of two of the methyoxy groups, the molecule is planar. The r.m.s. deviation through the 23 non-hydrogen and non-methoxy atoms is 0.0879 Å. The maximum deviations from this plane are 0.1219 (11) Å for the N2 atom and -0.1498 (11) for the C14 atom. The terminal carbon atoms, C17–C19, of the methoxy groups lie -0.0840 (17), 0.7910 (16) and -0.3504 (19) Å, respectively, out of the least-squares plane, indicating that the central methoxy group is almost orthogonal to the benzene ring to which it is connected with the C18—O2—C14—C13 torsion angle being 81.64 (15)°. The conformation about the N3═C10 bond [1.2829 (17) Å] is *E*.

In the crystal packing, weak N—H···O hydrogen bonds along with C—H···O interactions, Table 1, and π—π interactions between symmetry related quinolinyl residues [centroid···centroid distance = 3.4375 (8) Å for symmetry operation -1/2 + *x*, *y*, 1/2 - *z*] link molecules into a three-dimensional architecture. Globally, molecules stack along the *c* axis with alternating layers of quinolinyl and trimethoxybenzene residues, Fig. 2.

### Experimental

The compound was prepared from 7-chloro-4-quinolinylhydrazone and 3,4,5-trimethoxybenzaldehyde (Montenegro *et al.*, 2012) and was recrystallized from an EtOCH_2_CH_2_OH solution of the compound.

### Refinement

The C-bound H atoms were geometrically placed (C—H = 0.95–0.98 Å) and refined as riding with *U*_iso_(H) = 1.2–1.5*U*_eq_(C). The N-bound H-atom was located in a difference Fourier map and refined with N—H = 0.88±0.01 Å, and with *U*_iso_(H) = 1.2*U*_eq_(N). Owing to poor agreement, the (2 2 0), (2 3 0), (0 4 1), (2 2 1), (1 0 4), (2 1 2) and (1 0 2) reflections were omitted from the final cycles of refinement.

### Crystal data

**Table:**

C_19_H_18_ClN_3_O_3_	*F*(000) = 1552
*M**_r_* = 371.81	*D*_x_ = 1.447 Mg m^−^^3^
Orthorhombic, *P**b**c**a*	Mo *K*α radiation, λ = 0.71073 Å
Hall symbol: -P 2ac 2ab	Cell parameters from 24183 reflections
*a* = 7.6338 (2) Å	θ = 2.9–27.5°
*b* = 15.5335 (4) Å	µ = 0.25 mm^−^^1^
*c* = 28.7960 (7) Å	*T* = 120 K
*V* = 3414.62 (15) Å^3^	Prism, colourless
*Z* = 8	0.45 × 0.40 × 0.30 mm

### Data collection

**Table:**

Bruker–Nonius Roper CCD camera on a κ-goniostat diffractometer	3899 independent reflections
Radiation source: Bruker–Nonius FR591 rotating anode	3353 reflections with *I* > 2σ(*I*)
Graphite monochromator	*R*_int_ = 0.040
Detector resolution: 9.091 pixels mm^-1^	θ_max_ = 27.5°, θ_min_ = 3.0°
φ and ω scans	*h* = −7→9
Absorption correction: multi-scan (*SADABS*; Sheldrick, 2007)	*k* = −19→20
*T*_min_ = 0.652, *T*_max_ = 0.746	*l* = −26→37
22673 measured reflections	

### Refinement

**Table:**

Refinement on *F*^2^	Primary atom site location: structure-invariant direct methods
Least-squares matrix: full	Secondary atom site location: difference Fourier map
*R*[*F*^2^ > 2σ(*F*^2^)] = 0.036	Hydrogen site location: inferred from neighbouring sites
*wR*(*F*^2^) = 0.101	H atoms treated by a mixture of independent and constrained refinement
*S* = 1.02	*w* = 1/[σ^2^(*F*_o_^2^) + (0.0576*P*)^2^ + 1.3595*P*] where *P* = (*F*_o_^2^ + 2*F*_c_^2^)/3
3899 reflections	(Δ/σ)_max_ = 0.001
241 parameters	Δρ_max_ = 0.30 e Å^−^^3^
1 restraint	Δρ_min_ = −0.30 e Å^−^^3^

### Special details

**Table:**

Geometry. All s.u.'s (except the s.u. in the dihedral angle between two l.s. planes) are estimated using the full covariance matrix. The cell s.u.'s are taken into account individually in the estimation of s.u.'s in distances, angles and torsion angles; correlations between s.u.'s in cell parameters are only used when they are defined by crystal symmetry. An approximate (isotropic) treatment of cell s.u.'s is used for estimating s.u.'s involving l.s. planes.
Refinement. Refinement of *F*^2^ against ALL reflections. The weighted *R*-factor *wR* and goodness of fit *S* are based on *F*^2^, conventional *R*-factors *R* are based on *F*, with *F* set to zero for negative *F*^2^. The threshold expression of *F*^2^ > 2σ(*F*^2^) is used only for calculating *R*-factors(gt) *etc*. and is not relevant to the choice of reflections for refinement. *R*-factors based on *F*^2^ are statistically about twice as large as those based on *F*, and *R*- factors based on ALL data will be even larger.

### Fractional atomic coordinates and isotropic or equivalent isotropic displacement parameters (Å^2^)

**Table:**

	*x*	*y*	*z*	*U*_iso_*/*U*_eq_	
Cl1	0.32570 (5)	0.04598 (2)	0.339654 (12)	0.02558 (12)	
O1	−0.29202 (13)	0.51292 (6)	−0.01170 (3)	0.0189 (2)	
O2	−0.26948 (13)	0.42177 (6)	−0.09143 (3)	0.0185 (2)	
O3	−0.08960 (13)	0.27516 (6)	−0.09550 (3)	0.0185 (2)	
N1	0.03404 (15)	0.32333 (7)	0.28807 (4)	0.0185 (2)	
N2	0.06914 (15)	0.25871 (7)	0.14639 (4)	0.0166 (2)	
H2n	0.123 (2)	0.2115 (8)	0.1386 (5)	0.020*	
N3	−0.00092 (14)	0.30964 (7)	0.11233 (4)	0.0160 (2)	
C1	−0.02236 (18)	0.37484 (8)	0.25478 (5)	0.0183 (3)	
H1	−0.0738	0.4278	0.2641	0.022*	
C2	−0.01331 (18)	0.35863 (9)	0.20702 (5)	0.0168 (3)	
H2A	−0.0579	0.3991	0.1853	0.020*	
C3	0.06177 (17)	0.28249 (8)	0.19210 (4)	0.0144 (3)	
C4	0.12967 (17)	0.22434 (8)	0.22641 (4)	0.0141 (3)	
C5	0.21216 (17)	0.14533 (9)	0.21567 (5)	0.0167 (3)	
H5	0.2271	0.1295	0.1840	0.020*	
C6	0.27103 (18)	0.09103 (9)	0.24970 (5)	0.0177 (3)	
H6	0.3268	0.0383	0.2419	0.021*	
C7	0.24732 (18)	0.11490 (9)	0.29641 (5)	0.0174 (3)	
C8	0.16982 (18)	0.19071 (9)	0.30860 (5)	0.0179 (3)	
H8	0.1564	0.2052	0.3405	0.021*	
C9	0.10938 (17)	0.24777 (9)	0.27378 (4)	0.0152 (3)	
C10	0.01112 (17)	0.27774 (9)	0.07136 (5)	0.0157 (3)	
H10	0.0656	0.2231	0.0679	0.019*	
C11	−0.05455 (17)	0.32096 (9)	0.02980 (4)	0.0152 (3)	
C12	−0.14098 (17)	0.40041 (8)	0.03151 (4)	0.0155 (3)	
H12	−0.1551	0.4297	0.0602	0.019*	
C13	−0.20597 (17)	0.43605 (8)	−0.00934 (5)	0.0153 (3)	
C14	−0.18806 (17)	0.39204 (9)	−0.05162 (4)	0.0157 (3)	
C15	−0.09928 (17)	0.31355 (8)	−0.05291 (4)	0.0152 (3)	
C16	−0.03088 (18)	0.27845 (8)	−0.01241 (4)	0.0160 (3)	
H16	0.0318	0.2257	−0.0135	0.019*	
C17	−0.31426 (19)	0.55864 (9)	0.03114 (5)	0.0219 (3)	
H17A	−0.3887	0.5249	0.0521	0.033*	
H17B	−0.3696	0.6144	0.0250	0.033*	
H17C	−0.1997	0.5678	0.0456	0.033*	
C18	−0.1751 (2)	0.48981 (9)	−0.11456 (5)	0.0225 (3)	
H18A	−0.1514	0.5364	−0.0925	0.034*	
H18B	−0.2456	0.5120	−0.1404	0.034*	
H18C	−0.0641	0.4673	−0.1266	0.034*	
C19	−0.0276 (2)	0.18760 (9)	−0.09654 (5)	0.0228 (3)	
H19A	0.0954	0.1860	−0.0869	0.034*	
H19B	−0.0382	0.1648	−0.1282	0.034*	
H19C	−0.0979	0.1524	−0.0753	0.034*	

### Atomic displacement parameters (Å^2^)

**Table:**

	*U*^11^	*U*^22^	*U*^33^	*U*^12^	*U*^13^	*U*^23^
Cl1	0.0281 (2)	0.0275 (2)	0.02106 (19)	0.00361 (14)	−0.00295 (14)	0.00990 (14)
O1	0.0219 (5)	0.0175 (5)	0.0174 (5)	0.0038 (4)	−0.0027 (4)	0.0002 (4)
O2	0.0205 (5)	0.0199 (5)	0.0151 (5)	−0.0036 (4)	−0.0054 (4)	0.0060 (4)
O3	0.0240 (5)	0.0200 (5)	0.0115 (4)	0.0007 (4)	−0.0005 (4)	−0.0013 (4)
N1	0.0204 (6)	0.0187 (6)	0.0164 (5)	−0.0006 (5)	0.0001 (4)	−0.0014 (5)
N2	0.0216 (6)	0.0163 (5)	0.0120 (5)	0.0049 (5)	−0.0013 (4)	0.0017 (4)
N3	0.0168 (6)	0.0177 (5)	0.0135 (5)	−0.0003 (4)	−0.0015 (4)	0.0039 (4)
C1	0.0193 (7)	0.0155 (6)	0.0201 (7)	0.0008 (5)	0.0019 (5)	−0.0019 (5)
C2	0.0187 (7)	0.0154 (6)	0.0164 (6)	−0.0006 (5)	−0.0004 (5)	0.0022 (5)
C3	0.0133 (6)	0.0161 (6)	0.0139 (6)	−0.0025 (5)	0.0002 (5)	0.0008 (5)
C4	0.0133 (6)	0.0157 (6)	0.0133 (6)	−0.0033 (5)	−0.0001 (5)	0.0013 (5)
C5	0.0168 (6)	0.0185 (6)	0.0148 (6)	−0.0011 (5)	−0.0010 (5)	−0.0010 (5)
C6	0.0172 (7)	0.0151 (6)	0.0209 (7)	−0.0001 (5)	−0.0018 (5)	−0.0004 (5)
C7	0.0161 (6)	0.0191 (6)	0.0169 (6)	−0.0031 (5)	−0.0030 (5)	0.0057 (5)
C8	0.0186 (7)	0.0222 (7)	0.0128 (6)	−0.0024 (5)	0.0001 (5)	0.0005 (5)
C9	0.0139 (6)	0.0166 (6)	0.0151 (6)	−0.0028 (5)	0.0002 (5)	−0.0001 (5)
C10	0.0166 (6)	0.0154 (6)	0.0152 (6)	0.0000 (5)	−0.0006 (5)	0.0009 (5)
C11	0.0134 (6)	0.0184 (6)	0.0139 (6)	−0.0028 (5)	−0.0008 (5)	0.0019 (5)
C12	0.0162 (6)	0.0174 (6)	0.0129 (6)	−0.0025 (5)	−0.0012 (5)	−0.0005 (5)
C13	0.0137 (6)	0.0137 (6)	0.0184 (7)	−0.0018 (5)	−0.0009 (5)	0.0012 (5)
C14	0.0157 (6)	0.0182 (6)	0.0132 (6)	−0.0030 (5)	−0.0034 (5)	0.0035 (5)
C15	0.0165 (6)	0.0169 (6)	0.0123 (6)	−0.0040 (5)	0.0004 (5)	−0.0002 (5)
C16	0.0165 (6)	0.0158 (6)	0.0159 (6)	−0.0009 (5)	−0.0001 (5)	0.0019 (5)
C17	0.0241 (7)	0.0196 (7)	0.0218 (7)	0.0045 (6)	−0.0039 (6)	−0.0038 (6)
C18	0.0281 (8)	0.0202 (7)	0.0190 (7)	−0.0047 (6)	−0.0019 (6)	0.0062 (6)
C19	0.0316 (8)	0.0207 (7)	0.0162 (6)	0.0024 (6)	0.0010 (6)	−0.0032 (6)

### Geometric parameters (Å, º)

**Table:**

Cl1—C7	1.7478 (13)	C6—H6	0.9500
O1—C13	1.3646 (16)	C7—C8	1.364 (2)
O1—C17	1.4335 (17)	C8—C9	1.4156 (18)
O2—C14	1.3836 (15)	C8—H8	0.9500
O2—C18	1.4422 (16)	C10—C11	1.4609 (18)
O3—C15	1.3659 (15)	C10—H10	0.9500
O3—C19	1.4403 (17)	C11—C16	1.3949 (18)
N1—C1	1.3208 (18)	C11—C12	1.4003 (18)
N1—C9	1.3704 (17)	C12—C13	1.3915 (18)
N2—C3	1.3684 (16)	C12—H12	0.9500
N2—N3	1.3687 (15)	C13—C14	1.4030 (18)
N2—H2n	0.871 (9)	C14—C15	1.3954 (19)
N3—C10	1.2829 (17)	C15—C16	1.3893 (18)
C1—C2	1.4001 (19)	C16—H16	0.9500
C1—H1	0.9500	C17—H17A	0.9800
C2—C3	1.3826 (19)	C17—H17B	0.9800
C2—H2A	0.9500	C17—H17C	0.9800
C3—C4	1.4355 (18)	C18—H18A	0.9800
C4—C5	1.4136 (19)	C18—H18B	0.9800
C4—C9	1.4202 (17)	C18—H18C	0.9800
C5—C6	1.3689 (19)	C19—H19A	0.9800
C5—H5	0.9500	C19—H19B	0.9800
C6—C7	1.4068 (19)	C19—H19C	0.9800
			
C13—O1—C17	116.59 (10)	C11—C10—H10	118.3
C14—O2—C18	113.76 (10)	C16—C11—C12	120.60 (12)
C15—O3—C19	116.66 (10)	C16—C11—C10	116.86 (12)
C1—N1—C9	115.96 (12)	C12—C11—C10	122.53 (12)
C3—N2—N3	121.14 (11)	C13—C12—C11	119.26 (12)
C3—N2—H2n	119.7 (11)	C13—C12—H12	120.4
N3—N2—H2n	119.1 (11)	C11—C12—H12	120.4
C10—N3—N2	114.06 (11)	O1—C13—C12	124.19 (12)
N1—C1—C2	126.01 (13)	O1—C13—C14	115.47 (11)
N1—C1—H1	117.0	C12—C13—C14	120.33 (12)
C2—C1—H1	117.0	O2—C14—C15	119.20 (12)
C3—C2—C1	118.65 (12)	O2—C14—C13	120.84 (12)
C3—C2—H2A	120.7	C15—C14—C13	119.75 (12)
C1—C2—H2A	120.7	O3—C15—C16	124.20 (12)
N2—C3—C2	123.15 (12)	O3—C15—C14	115.58 (11)
N2—C3—C4	118.51 (12)	C16—C15—C14	120.21 (12)
C2—C3—C4	118.30 (12)	C15—C16—C11	119.79 (12)
C5—C4—C9	118.76 (12)	C15—C16—H16	120.1
C5—C4—C3	123.82 (12)	C11—C16—H16	120.1
C9—C4—C3	117.41 (12)	O1—C17—H17A	109.5
C6—C5—C4	121.64 (12)	O1—C17—H17B	109.5
C6—C5—H5	119.2	H17A—C17—H17B	109.5
C4—C5—H5	119.2	O1—C17—H17C	109.5
C5—C6—C7	118.67 (12)	H17A—C17—H17C	109.5
C5—C6—H6	120.7	H17B—C17—H17C	109.5
C7—C6—H6	120.7	O2—C18—H18A	109.5
C8—C7—C6	121.97 (12)	O2—C18—H18B	109.5
C8—C7—Cl1	119.60 (10)	H18A—C18—H18B	109.5
C6—C7—Cl1	118.40 (11)	O2—C18—H18C	109.5
C7—C8—C9	119.98 (12)	H18A—C18—H18C	109.5
C7—C8—H8	120.0	H18B—C18—H18C	109.5
C9—C8—H8	120.0	O3—C19—H19A	109.5
N1—C9—C8	117.41 (12)	O3—C19—H19B	109.5
N1—C9—C4	123.62 (12)	H19A—C19—H19B	109.5
C8—C9—C4	118.97 (12)	O3—C19—H19C	109.5
N3—C10—C11	123.46 (12)	H19A—C19—H19C	109.5
N3—C10—H10	118.3	H19B—C19—H19C	109.5
			
C3—N2—N3—C10	−177.97 (12)	N2—N3—C10—C11	−179.92 (12)
C9—N1—C1—C2	0.7 (2)	N3—C10—C11—C16	179.59 (12)
N1—C1—C2—C3	−0.4 (2)	N3—C10—C11—C12	−1.8 (2)
N3—N2—C3—C2	−0.9 (2)	C16—C11—C12—C13	1.09 (19)
N3—N2—C3—C4	176.90 (11)	C10—C11—C12—C13	−177.49 (12)
C1—C2—C3—N2	176.69 (12)	C17—O1—C13—C12	0.29 (18)
C1—C2—C3—C4	−1.07 (19)	C17—O1—C13—C14	179.15 (12)
N2—C3—C4—C5	3.29 (19)	C11—C12—C13—O1	−179.96 (12)
C2—C3—C4—C5	−178.84 (12)	C11—C12—C13—C14	1.23 (19)
N2—C3—C4—C9	−175.68 (11)	C18—O2—C14—C15	−103.67 (14)
C2—C3—C4—C9	2.19 (18)	C18—O2—C14—C13	81.64 (15)
C9—C4—C5—C6	0.58 (19)	O1—C13—C14—O2	−6.46 (18)
C3—C4—C5—C6	−178.38 (13)	C12—C13—C14—O2	172.44 (12)
C4—C5—C6—C7	0.3 (2)	O1—C13—C14—C15	178.87 (11)
C5—C6—C7—C8	−0.9 (2)	C12—C13—C14—C15	−2.22 (19)
C5—C6—C7—Cl1	−179.08 (10)	C19—O3—C15—C16	9.53 (19)
C6—C7—C8—C9	0.4 (2)	C19—O3—C15—C14	−169.05 (12)
Cl1—C7—C8—C9	178.63 (10)	O2—C14—C15—O3	4.77 (18)
C1—N1—C9—C8	−179.44 (12)	C13—C14—C15—O3	179.53 (11)
C1—N1—C9—C4	0.65 (19)	O2—C14—C15—C16	−173.87 (12)
C7—C8—C9—N1	−179.42 (12)	C13—C14—C15—C16	0.88 (19)
C7—C8—C9—C4	0.50 (19)	O3—C15—C16—C11	−177.10 (12)
C5—C4—C9—N1	178.92 (12)	C14—C15—C16—C11	1.4 (2)
C3—C4—C9—N1	−2.05 (19)	C12—C11—C16—C15	−2.4 (2)
C5—C4—C9—C8	−0.99 (18)	C10—C11—C16—C15	176.23 (12)
C3—C4—C9—C8	178.03 (12)		

### Hydrogen-bond geometry (Å, º)

**Table:**

*D*—H···*A*	*D*—H	H···*A*	*D*···*A*	*D*—H···*A*
N2—H2*n*···O3^i^	0.87 (1)	2.53 (2)	3.0349 (15)	118 (1)
C19—H19*B*···N1^ii^	0.98	2.48	3.3602 (18)	149

Symmetry codes: (i) *x*+1/2, −*y*+1/2, −*z*; (ii) *x*, −*y*+1/2, *z*−1/2.
